# Universal Versus Conditional Third Day Follow-Up Visit for Children With Nonsevere Unclassified Fever at the Community Level in Ethiopia: Protocol for a Cluster Randomized Noninferiority Trial

**DOI:** 10.2196/resprot.9780

**Published:** 2018-04-12

**Authors:** Karin Källander, Tobias Alfven, Ayalkibet Abebe Workineh, Abreham Hailemariam,, Max Petzold, Dawit Getachew, Lawrence Barat, Laura C Steinhardt, Julie R Gutman

**Affiliations:** ^1^ Malaria Consortium London United Kingdom; ^2^ Department of Public Health Sciences Karolinska Institutet Stockholm Sweden; ^3^ Sachs’ Children and Youth Hospital Stockholm Sweden; ^4^ Malaria Consortium Ethiopia Addis Ababa Ethiopia; ^5^ Health Metrics Sahlgrenska Academy University of Gothenburg Gothenburg Sweden; ^6^ School of Public Health Faculty of Health Sciences University of the Witwatersrand Johannesburg South Africa; ^7^ US President's Malaria Initiative United States Agency for International Development Washington, DC United States; ^8^ Malaria Branch US Centers for Disease Control and Prevention Atlanta, GA United States

**Keywords:** community health workers, Ethiopia, malaria, fever, child

## Abstract

**Background:**

Under the World Health Organization’s integrated community case management strategy, febrile children seen by community health workers (on day 1) without a diagnosable illness and without danger signs are advised to return on day 3, regardless of symptom resolution. This advice might be unnecessary and place additional time and cost burdens on caregivers and community health workers. However, the safety of not following up with respect to children with unclassified fever is unknown.

**Objective:**

The objective of this study is to establish the safety of conditional follow-up of nonsevere unclassified fever, that is, nonsevere illness with fever, no malaria, pneumonia, diarrhea, or danger signs, compared with universal follow-up on day 3, through a 2-arm cluster randomized controlled noninferiority trial.

**Methods:**

The study is being conducted in 3 districts in southwest Ethiopia. A total of 25 health facilities are randomized to one of the 2 intervention arms; all 144 health posts and 284 community health workers are included. All enrolled children are followed-up after 1 week (on day 8) for re-assessment. If still sick on day 8, additional follow-up takes place after 2 weeks (day 15) and 1 month (day 29). To demonstrate that there is no significant increase in the percentage of children deteriorating clinically, the sample size needed for a noninferiority margin of 4%, a power of 80%, an alpha of 5%, and a design effect of 3 is 4284 children with unclassified fever. Main outcome is treatment failure on day 8, defined as death, hospitalization, one or more danger signs, or persistent fever.

**Results:**

The project was funded in 2015 and enrollment was completed 2016. Data analysis is currently under way, and the first results are expected to be submitted for publication in 2018.

**Conclusions:**

This study addresses the question as to whether there is any benefit in recommending universal follow-up among children seen for nonsevere unclassified fever, or whether parents can be counseled to return in the event of persistent fever, using a cluster randomized controlled trial design embedded in a national program. Outcomes will be relevant for policy makers and are important for the evaluation of current and future World Health Organization guidelines for the management of children with fever.

**Trial Registration:**

ClinicalTrials.gov NCT02926625; https://clinicaltrials.gov/ct2/show/NCT02926625 (Archived by WebCite at http://www.webcitation.org/6xrQWn50t)

## Introduction

### Burden of Disease in Children in Sub-Saharan Africa

Even though substantial progress has been made in reducing child mortality around the world, many countries did not achieve the Millennium Development Goal 4 [1]. For countries to reach the Sustainable Development Goals by 2030 [2], it is crucial that child health interventions focus on management of infectious diseases in children. Globally, mortality in children under 5 years stands at 43 per 1000 live births; it is estimated that 5.6 million children under 5 years die each year [3,4]. A large proportion of deaths are caused by infectious diseases such as pneumonia (15.5%), diarrhea (8.9%), and malaria (5.2%)—diseases with symptoms that overlap, making differential diagnosis difficult [5]. In response, many countries in sub-Saharan Africa have introduced integrated community case management (iCCM), where community health workers (CHWs) are trained to assess, classify, and treat uncomplicated cases of pneumonia, diarrhea, and malaria in children U5, and refer children with danger signs and malnutrition for facility-based care [6]. Although the mortality impact of iCCM has been difficult to demonstrate [7], there is clear evidence that it can increase the treatment rate among sick children [8].

### Control Strategies for Childhood Illnesses

In Ethiopia, the under-five mortality rate stands at 58 per 1000 live births; it is estimated that 187,000 children under 5 years died in the year 2016 [4,9]. As part of Ethiopia’s Health Extension Program, the Government has deployed over 42,000 female CHWs, or Health Extension Workers (HEWs) [10,11], to provide preventive, promotive, and curative health services at the community level; since 2010, iCCM has been scaled up in most regions of the country. There are typically 2 HEWs assigned to a health post in a sub-district with a population of 3000-5000. The HEWs are supervised by health centers that oversee approximately 5 health posts each. Medicines and supplies are distributed to the health posts by the Federal Ministry of Health, Regional Health Bureaus, and woreda (district) health offices, as well as by implementing partners. On the basis of surveys conducted in 2012, 90% of HEWs had all the essential iCCM drugs and supplies [12], and HEWs provided correct case management for 64% of children [13].

As per the World Health Organization’s iCCM guidelines [14], children diagnosed by a CHW with an illness are given treatment (on day 1) and counseled to return on day 3 to assess treatment compliance and illness resolution. Children with fever but without a diagnosable illness and without danger signs (ie, nonsevere unclassified fever) for whom treatment should be withheld are also told to return to the CHW on day 3, even if the child has recovered. This *universal* follow-up visit is either done through a return visit to the health post or through a home visit by the CHW.

### Unclassified Fever in Children

However, febrile illness is common in childhood, and is often due to viruses or other self-resolving illnesses [15,16]. In a large proportion of cases, fever resolves rapidly, almost always within 96 hours [17]. A number of studies have suggested that it is safe to withhold medical treatment for children with unclassified fever [18]. In Ethiopia, HEWs are instructed to follow the integrated management of neonatal and childhood illness (IMNCI) manual, which recommends that children seen by HEWs should only return for a re-assessment if the illness persists or deteriorates, that is, a *conditional* follow-up visit to the health post. However, HEWs and their supervisors report that a range of practices are applied for children with unclassified fever, including both conditional (as recommended) and universal follow-up advice, immediate referral to health centers, or treatment with antimalarial tablets, despite a negative malaria Rapid Diagnostic Test (mRDT).

There is limited evidence on which of the 2 follow-up recommendations (conditional as in IMNCI or universal as in iCCM) is safer for the child, and it is unclear whether caregivers of children actually come back promptly to the health post for their conditional follow-up visit if the child is not improving, or if they come back at all if the child in a universal follow-up situation has improved. Bacterial infections can develop quickly, and delaying care-seeking is a major risk factor for death in both pneumonia and malaria [19,20]; hence, children with untreated persistent fever may be at risk if caregivers do not comply with the conditional follow-up advice. A universal follow-up visit 2 days after an initial assessment for all children may promote detection of those at risk of developing severe illness. However, it could also potentially lead to delayed care-seeking for children whose health rapidly deteriorates at home if caregivers instead wait for their booked follow-up visit. In addition, the visit may add extra burden to families and HEWs and might be unnecessary if fever has resolved. On the caregiver side, opportunity costs and other barriers often hinder care-seeking for sick children, even when community-based providers are near and free of charge [21]. It is therefore unclear whether caregivers and HEWs would comply better with the conditional follow-up advice compared with the universal follow-up advice and whether the universal follow-up visit is even necessary.

This paper presents a protocol for a 2-arm cluster randomized controlled noninferiority trial conducted to assess whether conditional follow-up is noninferior to universal follow-up for nonsevere febrile illness in children U5, in whom malaria, pneumonia, diarrhea, or danger signs are absent.

## Methods

### Study Aim and Objectives

The study aims to assess the safety, in terms of the proportion of children whose health clinically deteriorates, of a follow-up visit conditional on nonresolution of symptoms for mRDT-negative children with no fast breathing, pneumonia, diarrhea, or danger signs, managed at the community level, compared with a universal visit for all these children on day 3.

#### Primary Objectives

The primary objective of the study was to assess the treatment failure after 1 week (on day 8), defined as the proportion of children with nonsevere unclassified fever who subsequently declined clinically (death, hospitalization, one or more danger signs, or persistent fever) subsequent to (1) conditional versus (2) universal follow-up of children under 5 years who present to HEWs with unclassified, nonsevere fever in 3 woredas (districts) in the Southern Nations, Nationalities and People’s Region (SNNPR) in southwest Ethiopia.

#### Secondary Objectives

The secondary objectives of this study were as follows:

1. To describe the clinical presentation and outcome of illness in those children whose symptoms do not resolve or where danger signs develop at day 8, to measure the rate of treatment failure at day 15 and 29 in both study arms for children who did not recover by day 8, and to determine the percentage of children who return for scheduled visits on day 3 (universal arm) or spontaneous visits before day 8 (universal and conditional arms).
2. To determine the percentage of secondary treatment (antimicrobial medicines prescribed during visits to any providers after initial presentation to HEWs) in both study arms on day 8.
3. To assess acceptability of the conditional or universal follow-up recommendations and no treatment with an antimicrobial to caretakers and HEWs, and determine why caretakers chose to return or not return to the HEW.

### Study Design

This is a 2-arm cluster randomized controlled trial (cRCT) carried out in 3 woredas in SNNPR in Ethiopia. Clusters defined by the health center (the lowest administrative unit where HEW services are coordinated) are randomized into either the *conditional* or *universal* follow-up arm. All children seeking care from the HEW health posts in these clusters are potential recipients of the interventions, in addition to having access to routine care available from private and public health services. Caregivers of children who meet the inclusion criteria (fever without malaria, pneumonia, diarrhea, or other symptoms requiring referral) are counseled to follow 1 of the 2 pathways, based on which intervention cluster the HEW belongs to. There are 25 clusters; 13 clusters in the universal follow-up arm and 12 in the conditional follow-up arm.

### Study Site

**Figure 1:**
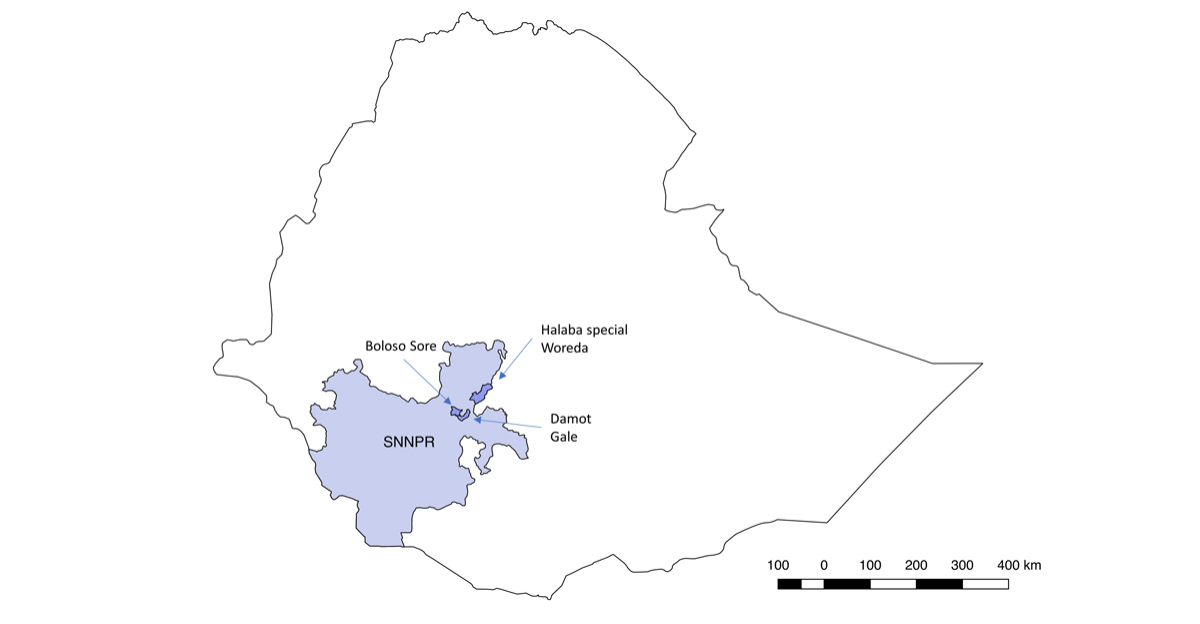
Map of Ethiopia with the Southern Nations, Nationalities and People’s Region (SNNPR) and the three study woredas.

This research study will be conducted in 3 woredas , namely, Boloso Sore, Damot Gale, and Halaba in the Wolayita zone of SNNPR in southwest Ethiopia (see Figure 1). The iCCM program is functioning in all districts of SNNPR through support to the Regional Health Bureau from Save the Children International and the Integrated Family Health Program. It will therefore be an ideal environment to implement this research, as iCCM services are stable and the program is implemented by the Regional Health Bureau, which will provide technical oversight to this project.

According to the latest malaria indicator surveys, the rate of prompt care-seeking for fever is currently low, with only 46.3% of children under 5 years with fever taken for early treatment [22]. However, care-seeking at health post level shows an upward trend, presumably as a result of the increased awareness of the availability and proximity of child health services [12]. In addition, Malaria Consortium, with funding from the James Percy Foundation, has recently started implementing the Integrated Community-based Interventions for Malaria Services project in SNNPR. Activities include case detection by the volunteer Health Development Army who will work to ensure that all children with fever receive prompt diagnosis and treatment, and that children with danger signs get referred. As part of this grant, refresher training will also be provided to HEWs to negotiate optimal practices using behavior change communication tools and facilitation skills in community conversation. It is anticipated that these changes will lead to an increased use of HEWs in the study area.

The 3 woredas will be selected based on: (1) strength of iCCM program (ie, consistency in HEW supervision and supply), (2) HEW use rate among caregivers (≥50 children assessed for fever each month over a 12-month period), and (3) concurrent community mobilization activities under other grants (to ensure that demand was kept high during the study period). There are 25 health centers and 144 health posts with 284 HEWs in the 3 selected woredas.

### The Interventions

**Figure 2:**
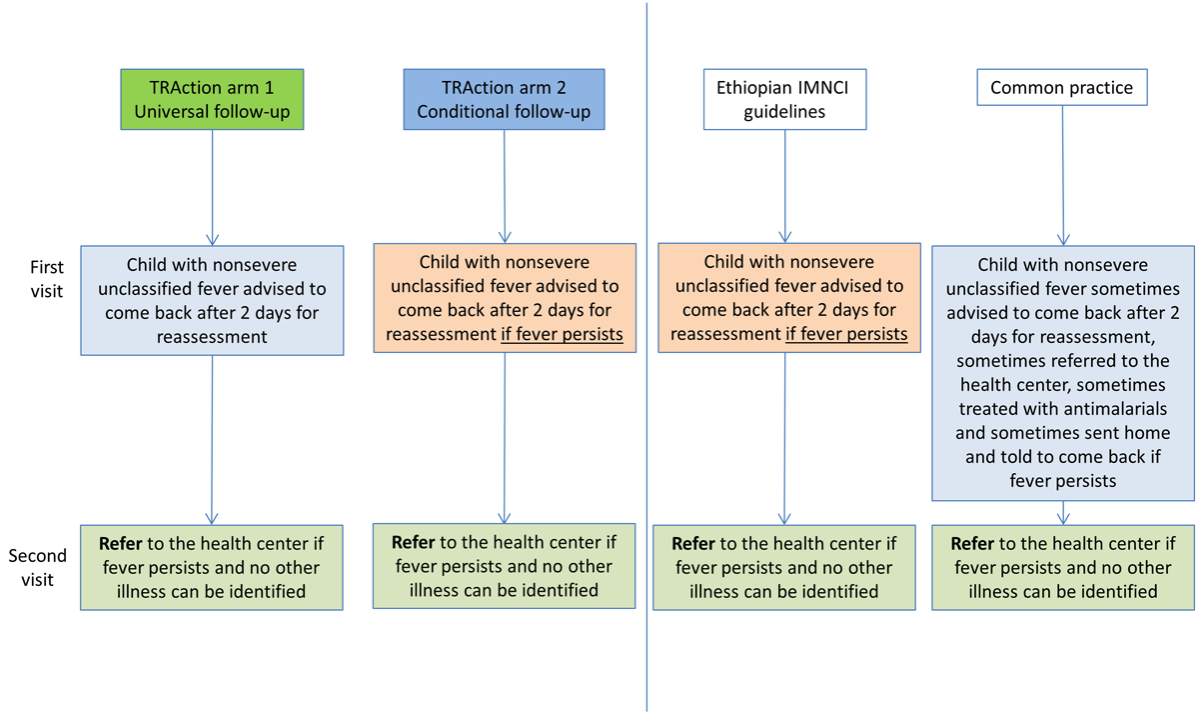
Description of the 2 interventions arms, the current guidelines, and observed common practice. IMNCI: integrated management of neonatal and childhood illness.

Children aged 2-59 months with fever (≥37.5 degrees Celsius) or a history of fever, a negative mRDT, no other symptoms of pneumonia or diarrhea, and no danger signs will be eligible to participate in the study. Figure 2 outlines the areas that consenting caregivers will be counseled on how to detect danger signs and seek care immediately from a health center if danger signs develop or the illness worsens; fever reduction strategies, such as tepid sponging and paracetamol; and that a study visit to assess clinical outcomes will take place after 1 week. Caregivers in the conditional arm will also be advised to return at any point to the HEW at the health post for re-assessment if symptoms persist or deteriorate (as per the Ethiopian IMNCI Guidelines), whereas caregivers in the conditional arm will be advised to return on day 3 to the HEW for a follow-up assessment, even if the child has recovered (as is common practice).

At the day 3 re-assessment visit in the universal follow-up arm and at any spontaneous visit in both arms, the child will have a full re-assessment of their condition and the HEW will fill out a child assessment form. Caregivers will be asked whether the child remains febrile or whether the illness has resolved. If the child still has unclassified fever and a negative mRDT on re-assessment, the child will be referred to the nearest health center, as recommended in the national IMNCI guidelines. If the illness has resolved, the child will be sent home.

A clinically trained independent evaluator (IE) who is blinded to the study arm will visit all enrolled children at their home after 1 week to assess their clinical outcome. If caregivers report that the child is no longer ill and no fever is recorded, the child will be considered cured and no more follow-up will be done. If the caregiver reports that the child still has symptoms or if fever or other illness symptoms are detected during the assessment, the IE will follow the IMNCI algorithm and refer/treat the child accordingly. The IE will then follow-up again via a home visit after 2 weeks (day 15) and, if the child is still ill, on day 29.

At the day 8 visit, the IE will use a questionnaire to ask about individual and household characteristics, care-seeking and other treatments for the current illness episode, and reasons for returning/not returning to the HEW for follow-up care.

### Qualitative Component

Caregivers’ recognition and responses to childhood fevers and HEWs’ views and experiences of their position in the health care system during previous and, in particular, the current recommendations in the respective intervention arms will be explored using semistructured interviews at a time point when the interventions are fully adopted by the HEWs (determined based on a stable enrollment rate). A subset of mothers and HEWs in both arms will be selected for inclusion in these interviews to help put the findings into context.

### Randomization

Cluster randomization will be at the health center level, corresponding to the lowest administrative unit where HEW services are coordinated; there will be 25 clusters in the 3 study woredas, with an average of 5 health posts and 7.5 HEWs per cluster. All clusters will be eligible for randomization. Restricted randomization will be performed to minimize the difference between intervention and control arms on key indicators, including average under-five population size, cluster distance to nearest zonal referral hospital, and number of unclassified fevers in children under 5 years seen by HEWs [23]. A validity matrix will be produced to confirm that no pairs are more or less likely to appear together than they would by chance. Sorting of clusters and random selection of schemes will be carried out in STATA 13 (STATA Corp, College Station, TX, USA).

### Sample Size

The primary outcome on which sample size is based is the percentage of children with persistent fever, illness, or decline (hospital, danger signs develop, or death) at day 8. It is assumed that about 5% of children in both groups will still be ill at day 8 (based on rates of ~3% and 8% in previous studies [16,24]) and that this percentage will be approximately equivalent between groups. To calculate sample size, the outcome rate (ill at day 8) in the universal follow-up group is set to 5%, and it is assumed that the (true) corresponding outcome percentage in the conditional follow-up group will be no more than 6%. For the purpose of concluding that the conditional follow-up is noninferior to the universal follow-up approach, the upper bound of a one-sided 95% CI around the absolute difference in outcome rate (conditional minus universal) must not exceed 4% (noninferiority margin), assuming a power of 80% and an alpha of 5%. A design effect of 3 is used to account for clustering at HEW and health facility levels, generating a total sample size of 4284 children, with 2142 in each arm. To compensate for 10% loss to follow-up at day 8 and an additional 5% loss between day 8 and day 15, a total of 4900 children will be enrolled. Enrollment will occur over a period of 1 year to account for seasonality of various causes of febrile illness, starting in December 2015 and is expected to be completed around December 2016.

### Outcomes

All enrolled children will have a study visit in their home with an IE after 1 week to assess their clinical outcome. Children who have not recovered on day 8 will be re-assessed after 2 weeks, and those whose illness persists on day 15 will be re-assessed on day 29. In addition, all enrolled children will be followed-up via a phone call for vital status on day 28. Management of illness at any follow-up visit (ie, return to HEW on any day; return to HEW for universal day 3 visit; or day 8, 15, and 29 assessment) will follow established national IMNCI guidelines.

The primary outcome is treatment failure on day 8, defined as the proportion of children with unclassified fever who subsequently declined clinically (death, hospitalization, one or more danger signs or persistent fever).

Secondary outcomes include:

- Clinical presentation in those with unresolved illness at day 8 in each arm
- Treatment failure on day 15 and 29, defined as the proportion of children with unclassified fever who subsequently declined clinically (death, hospitalization, one or more danger signs or persistent fever)
- Percentage of children who present to the HEW for the follow-up visit on day 3 in the universal follow-up arm
- Percentage of children who spontaneously represent to HEW for persistence or worsening of symptoms in the conditional follow-up arm, and the timing of these visits
- Percentage of children receiving secondary treatment (antimicrobial medicines prescribed during visits to any providers after initial presentation to HEW) in each arm between enrollment and day 8
- Caregiver and HEW acceptability of universal and conditional follow-up recommendations

### Data Collection

HEWs will collect data using an Open Data Kit (ODK) [25] data collection form on mobile phones. Data will include date of enrollment, child identifiers, and clinical indicators (fever/axillary temperature, cough, respiratory rate, diarrhea, and danger signs). The enrollment data will be synchronized with a server which is accessed by a data manager who will download enrollments on a daily basis and schedule follow-up visits for 6 IEs using an online Google calendar.

The IEs will be equipped with tablets programmed with 3 ODK data collection forms; one for the day 8 visit, one for any extra visits (on day 15 or 29), and one vital status form for day 29. The data that will be collected during the household follow-up visits on day 8, 15, and 29 will include clinical data for the children following the IMNCI algorithm (eg, fever/axillary temperature, cough, respiratory rate, diarrhea, mid-upper arm circumference (MUAC) measure, and danger signs), any secondary treatment (antimicrobial medicines prescribed during visits to any providers after initial presentation to HEWs), hospitalization, care-seeking history, and costs, as well as caregiver and household characteristics. For children who cannot be found at home at the time of the home visit, 2 more attempts will be made over the 2 following days. After this, the child will be registered as a loss-to-follow-up for the primary outcome.

Moreover, 3 research assistants will enter data collected by HEWs for children who come back to the HEW spontaneously (universal and conditional arm) or on day 3 (universal arm) into an ODK sick child assessment form every 2 weeks.

A rigorous monitoring system will be implemented by the study team and will be part of the continuous quality assurance. The data manager will review forms submitted to the server daily and check for duplicates, completeness, and accuracy before storing them in the project database. Discrepancies, overdue follow-up visits, and other issues will be resolved by phone calls to the IEs and during weekly supervision meetings with field research staff. Biweekly field supervision visits to all HEWs will be carried out, and district HEW supervisors will be trained to monitor HEW trial activities during routine weekly group supervisions. A minimum of 10% of all enrolled cases and 50% of children with treatment failure will have a quality control re-assessment by a research assistant. The final dataset will be exported to STATA 13.

Semistructured interviews with HEWs and caregivers of children enrolled in the study will be conducted when a stable enrollment rate has been established in the study (assumed to be after 3 months). HEW interviews focus on what decentralization of health care mean to HEWs, how changes in follow-up recommendations are perceived, how they describe their roles in the communities, and how health system changes affect this role and their work situation. Caregiver interviews will aim to improve the understanding of caregiver perceptions of childhood illnesses, their perceived causes, and treatment options in the 3 woredas. Interview guides will capture how caregivers (assume primarily mothers) of sick children experience illness episodes and treatment seeking inside and outside the household. Focus will be on how caregivers describe the illness episode from the beginning to the recovery, current health status, actions that are taken or not taken when a young child gets fever, who is involved in the care of the child, and what their perceptions and experiences are with the recommended follow-up action they have been exposed to when visiting the HEW. Half of the HEWs and mothers to be interviewed will be from the study arm using the universal follow-up advice and the other half will follow the conditional follow-up recommendation. HEWs will be purposively selected, based on who has enrolled the highest number of children for the cRCT. Mothers of children enrolled in the study in the 2 weeks preceding the start of the qualitative data collection will be included using simple random sampling. A minimum of 1 week should have passed since the day 8 visit was completed to avoid study fatigue.

Interview guides will be separately prepared for the HEW and caregiver interview and translated into Amharic. In addition, 2 male, Amharic-speaking interviewers will conduct the HEW interviews and 1 additional male Amharic interviewer will be recruited for the caregiver interviews.

### Data Analyses

The analysis of the primary outcome, treatment failure on day 8, will be done following a per-protocol approach. The baseline characteristics of the children and caregivers in both arms will be summarized using descriptive statistics. The difference between the percentage of the main study outcomes (persistent fever, persistent illness, or clinical deterioration [mortality, hospitalization, or danger signs], and these outcomes combined) of the 2 follow-up arms will be calculated. If the upper bound of the 95% CI of the difference in this percentage is less than 4%, the null hypothesis will be rejected; the conclusion will be that the conditional follow-up approach is not inferior to the standard approach. If there are imbalances in baseline characteristics across the 2 intervention arms, adjusted analyses of the difference in percentages will be conducted using multivariate logistic regression models.

The primary outcome will be compared between groups using generalized linear models with a binomial distribution and identity link using a robust variance estimator, treating cluster as a random effect. We will apply a conventional statistical noninferiority test using a CI approach using the exact binomial CI for the difference in overall treatment failure between intervention arms. The main analysis will be done using the per-protocol population (only including children for whom the primary outcome was collected on day 8±1 and whose caregiver reported receiving follow-up advice from the HEW that was aligned with the study arm), as is appropriate for noninferiority and equivalence studies, together with sensitivity analysis in the per-protocol and intention-to-treat populations [26]. Other descriptive statistics, such as cost of care seeking and compliance with the intervention protocol, will be compared between study arms using *t test* and chi-square statistics, respectively.

Qualitative interview transcripts from HEWs and caregivers will be coded and merged into sub-categories, categories, and themes using Nvivo (NVivo qualitative data analysis Software; QSR International Pty Ltd. Version 11, 2015). HEW interviews will be analyzed first and its results reviewed after caregiver interviews are read. For the purpose of this study, caregiver interviews will be analyzed and compared with main themes identified in the HEW interviews.

Interim data analysis reports are produced on a quarterly basis to document cluster-specific cumulative enrollment rates, adherence to follow-up recommendations, and characteristics of the children and households enrolled. Deviance from the study protocol, or in expected enrollment rates, is acted on by providing refresher training and supervision to HEWs and research staff.

### Ethics Approval and Consent to Participate

The trial protocol was approved by the SNNPR State Health Bureau on September 23, 2015 (ref P026-19/4511). In addition, approval was obtained from the district authorities and local leaders in the woredas where the study is being conducted. Co-investigators from Centers for Disease Control and Prevention participated under a nonengaged determination from their Human Research Protection Office.

Each caregiver whose child is eligible for enrollment is provided with a sheet containing information about the study in Amharic. Caregivers are given time to read the information sheet, and the HEW verbally summarizes key points and answers any questions. Agreement to participate is indicated by signature or thumb print. The individual’s right to refuse consent or to stop the interview at any time after consent has been given is preserved.

The project has a 3-person Data Monitoring Committee (DMC) with external members having expertise in child health, epidemiology, iCCM, and randomized controlled trials. The DMC is responsible for safeguarding the interests of study participants, assessing the safety of the intervention during the trial, and for monitoring the overall conduct of the study. The DMC provides recommendations about stopping or continuing the study and other aspects of trial implementation, using interim data analysis reports on cluster-specific enrollment rates, adherence to follow-up recommendations, and on characteristics of the children and households enrolled.

The DMC is advisory to the study Steering Committee, which comprises the lead study investigators, who are jointly responsible for the design, conduct, and analysis of the trial. The Steering Committee is responsible for promptly reviewing the DMC recommendations, to decide whether to continue or terminate the study, and to determine whether amendments to the protocol or changes in study conduct are required.

## Results

The project was funded in 2015, and enrollment was completed in 2016. Data analysis is currently under way, and the first results are expected to be submitted for publication in 2018.

## Discussion

Febrile illnesses are among the leading causes of deaths in children U5. There is limited evidence on the safest and most efficient approach to manage unclassified fevers at the community level, and current practice is not always in line with the recommended guidelines. This cluster randomized controlled noninferiority trial aims to address this question by comparing universal follow-up recommendations, which are currently recommended for community management of sick children by WHO, with conditional follow-up recommendations, which are currently the guidelines provided to HEWs in Ethiopia, for children with nonsevere, unclassified fever.

The main strength of this study is the embedding of a robust randomized controlled trial into a national program context. An additional strength was the multidisciplinary research team, which had strong involvement of local researchers and Ministry of Health staff, and which was advised by a data monitoring committee comprising global child health epidemiologists. This constellation enables the application of robust methods for both quantitative and qualitative evaluation, as well as high-quality implementation, while creating evidence with greater potential for direct policy influence.

A potential limitation of this study is the generalizability of the findings, given that caregivers’ knowledge of the upcoming study visit could affect their care-seeking behavior. However, although the caregivers in both arms are informed that a research team member will come to their household in the next 4 weeks, they are not told which day they will expect the visit by the study team. Another possible limitation is that the qualitative interviews will all be done by men, whereas all the interviewees (HEWs and caregivers) are women. Although we attempted to recruit female qualitative research assistants, it was not possible to find women with the experience required, including fluency in the 3 local languages spoken in the region. To compensate for this, we will address this issue during interviews, as well as on pilot testing of the interview guides, to ensure that any gender-related issues will be prevented. This study, along with a sister study in Democratic Republic of Congo [27], is the only study designed to look at this critical policy issue. It therefore has high potential to contribute to the knowledge base by assessing and evaluating recommended practices for treating febrile children whose illness cannot be diagnosed at the community level using iCCM guidelines. Research outcomes from this study are relevant for both local and international policy makers, as they will provide an evaluation of current guidelines as well as information for the development of future World Health Organization guidelines.

This study is designed to directly influence policy and practice, especially for government-led implementation of iCCM in Ethiopia and other sub-Saharan African countries. Implementation has been carried out in close consultation with policy makers. During the early stages of the project, a communications and research uptake strategy was developed, which aims to increase the likelihood of the results being used to influence policy and practice by engaging with stakeholders throughout the research process. A sensitization workshop was conducted at the beginning of the project to ensure that stakeholders are involved in the technical aspects of the project from the beginning. HEWs were involved in the consultations throughout the study, and their opinions and experiences with the 2 follow-up recommendations, which were collected during the qualitative interviews, were discussed in depth in several of the dissemination events. An in-country technical advisor has agreed to support the team on this project by bridging the research results to planning and policy functions in the Federal Ministry of Health. Further anticipated communication outputs include a stakeholder report, a policy brief, a peer-reviewed publication of results, national- and regional-level dissemination meetings, and presentation in regional and international conferences. Results of this research will be shared with national decision makers, program implementers, HEWs, researchers, and other stakeholders to promote learning and inform potential modification of iCCM and the Integrated Management of Childhood Illness Guidelines.

## Abbreviations

CHW: community health workers
DMC: Data Monitoring Committee
HEW: health extension workers
iCCM: integrated community case management
IE: independent evaluator
IMNCI: integrated management of neonatal and childhood illness
mRDT: malaria Rapid Diagnostic Test
ODK: Open Data Kit
SNNPR: Southern Nations, Nationalities and People’s Region
